# The Role of CXCL13 in Antibody Responses to HIV-1 Infection and Vaccination

**DOI:** 10.3389/fimmu.2021.638872

**Published:** 2021-02-25

**Authors:** Yonas Bekele Feyissa, Francesca Chiodi, Yongjun Sui, Jay A. Berzofsky

**Affiliations:** ^1^ Vaccine Branch, Center for Cancer Research, National Cancer Institute, National Institutes of Health, Bethesda, MD, United States; ^2^ Department of Microbiology, Tumor and Cell Biology, Biomedicum, Karolinska Institutet, Solna, Sweden

**Keywords:** CXCL13, HIV-1, vaccine, CXCR5, broadly neutralizing antibodies

## Abstract

CXCL13 signals through the G protein-coupled chemokine receptor CXCR5 to drive development of secondary lymphoid tissue as well as B cell and Tfh cell trafficking to germinal centers (GC), which leads to the differentiation of B cells to plasma cells and memory B cells. CXCL13 has been proposed as a general plasma biomarker for GC activities. In HIV-1 infected individuals, plasma CXCL13 levels have been associated with the rate of disease progression to AIDS. Moreover, CXCL13 production has been reported to be increased in HIV-1-infected lymph nodes, which may drive increased downregulation of CXCR5. In this review, we address the role of CXCL13 in HIV-1 infected individuals with regard to GC formation, generation of broadly neutralizing antibodies after infection and vaccination, and AIDS-related B cell lymphoma.

## Introduction

CXCL13 (C-X-C motif chemokine ligand 13), originally known as B cell-attracting chemokine (BCA)-1 and B lymphocyte chemoattractant (BLC), is expressed in the liver, spleen, lymph nodes, appendix and stomach by follicular dendritic cells (FDCs), stromal cells, monocytes and macrophages (1–3). CXCL13 is the sole ligand for the 7 transmembrane-domain G protein-coupled chemokine receptor CXCR5, which was originally cloned from a Burkitt’s lymphoma cDNA library and named BLR-1 (Burkitt`s lymphoma receptor-1) (4). CXCR5 is expressed by B cells, CD4^+^, and CD8^+^ T cells, T regulatory cells and dendritic cells (DCs) (5–9).

CXCL13 plays an important role in lymphoid tissue development. Lymphoid tissue inducer cells are attracted by both CXCL13 and the CCR7-specific chemokine ligand CCL21 at an early phase of secondary lymphoid tissue development. CXCR5- and CXCL13-deficient mice both fail to cluster lymphoid tissue inducer cells to form an initial stage of lymph node development (10). CXCL13 also attracts B cells to secondary lymphoid tissue and is important for compartmentalization of germinal centers, where B cells undergo somatic hypermutation, affinity maturation and class switching (2).

**Figure 1:**
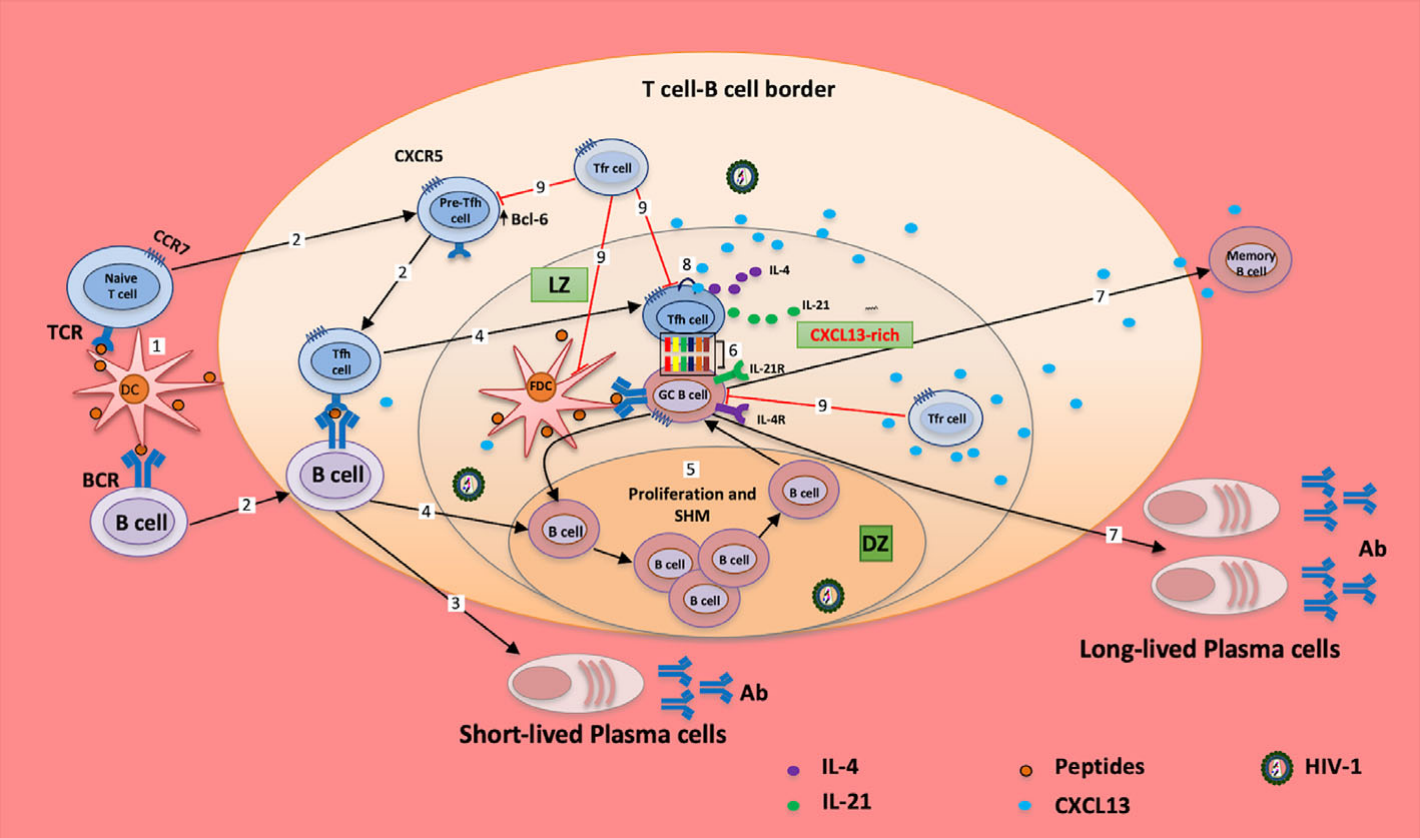
Tfh cells and CXCL13 in chronically HIV-1 infected individuals. Peptides (from vaccine/Ag)-loaded DCs migrate from the periphery to tissues/GC; cognate interaction of DCs with naïve/memory T cells and naïve/memory B cells induces the expression of BCL6, which in turn downregulates the expression of CCR7 and upregulates the expression of CXCR5 (1); cells migrate to the T cell-B cell border (2) for maturation and generation of short-lived plasma cells (3). B cells migrate to the dark zone of the GC (4); and undergo proliferation and somatic hypermutation (SHM) before migrating to the light zone (LZ) of the GC (5). Cellular interaction of GC B cells with Tfh cells (6) induces the production of long-lived plasma cells and memory B cells, and mediates somatic hypermutation, affinity maturation and class switch (7). However, excessive CXCL13 release from cells downregulates the expression CXCR5 in Tfh and B cells through autocrine/paracrine signaling (8), which impairs the successful interactions of these cells (6). Moreover, T follicular regulatory (Tfr) cells (9) regulate GC B cells, FDC and Tfh cell activities in the GC; it is evident that altered frequency of Tfr cells may account for the poor interaction of the GC B and Tfh cells observed in HIV-1 infection. These may increase the proportion of autoreactive Abs, exhausted B cells and poor response to vaccines/Ags, and collectively, may cause impairment of humoral responses (7). Ag, antigen; TCR, T cell receptor; BCR, B cell receptor; Tfh, T follicular helper cell; GC, germinal center; LZ, light zone; DZ, dark zone; SHM, somatic hypermutation; Tfr, T follicular regulatory; Ab, antibody.

CXCR5 expression by mature B cells and T follicular helper (Tfh) cells promotes their localization to the light zone of the germinal center (GC), where CXCL13 is concentrated (2) (**Figure 1**). B cells concentrated ectopically in the dark zone, when CXCR5-deficient B cells were transferred to B cell-deficient mice (11), and CXCR5-deficient mice presented with an altered structure of Peyer’s patches and primary splenic follicles (12). After DNP-KLH immunization, the B:T cell ratio was increased in the spleen in both wild type (WT) and CXCR5 knockout (KO) mice; however, in the periphery, the B:T cell ratio was reduced in WT mice and increased in the CXCR5 KO mice suggesting re-circulation of B cells out of the defective lymphoid tissues in the KO mice (12). The organization of B cells in the lymphoid follicles was affected in CXCL13-deficient mice with poor demarcation of the T-B cell zones (13).

CXCL13 has been linked to multiple biological processes, including GC activity after vaccination, protective antibody production, autoimmune disease and hematologic neoplasia. With regard to vaccination, studies have reported increased plasma CXCL13 levels, suggesting a potential role as a biomarker of GC activity. For example, 17D yellow fever vaccine and HIV-1 vaccine both induce robust immune responses and were both associated with significant increases of plasma CXCL13 levels at 7 days post-vaccination in adults (14). CXCL13 levels were significantly associated with antibody concentration and with the frequency of GC Tfh cells in both 17D yellow fever and HIV-1 vaccine recipients (14). 17D yellow fever vaccine is a live attenuated vaccine that induces a robust virus-specific CD8 T cell and neutralizing antibody response that lasts for decades. The HIV-1 vaccine tested was vectored by a live adenovirus, which may have limited the immune response in vaccinees having pre-vaccination antibodies from antecedent natural adenovirus infection. Studies on inactivated influenza vaccination showed that elevated plasma levels of CXCL13 were also detected at different time points in children; however, there was no correlation with GC activity (15, 16). The immune response to Trivalent inactivated influenza vaccine (TIV) was poor compared to the response to live attenuated influenza vaccine (LAIV) (17). Overall, the results were consistent with, but not proof of, the conclusion that live viral vaccines induce much more CXCL13 than subunit vaccines.

Efficient and direct study of the GC reaction after vaccination is not feasible in humans, which identifies a need for a reliable plasma biomarker (14). There is an extensive literature on CXCL13 and autoimmunity. A study conducted in rheumatoid arthritis (RA) patients showed that CXCL13 produced by follicular dendritic cells (FDCs) attracted B cells to the tertiary lymphoid follicles of the inflammatory sites (18). In mice transgenic for CXCL13 expression, more B cells were recruited to the thymus, which triggered inflammatory responses after Poly (I:C) injection; these mice were also more susceptible to myasthenia gravis upon immunization with Torpedo acetylcholine receptor (T-AChR) emulsified with complete Freund´s adjuvant (CFA) compared to WT mice (19). The migration of B cells was significantly diminished in CXCR5-deficient B6/lpr mice, a model for autoimmunity including systemic lupus erythematosus. However, the frequencies of B cells, double negative (CD4^-^CD8^-^) T cells and IL-17+ double negative T cells were elevated in the B6/lpr mice compared to CXCR5 deficient B6/lpr mice (20), suggesting the CXCL13-CXCR5 axis may also be involved in the recruitment of cells to the inflamed tissues.

Given the fundamental importance of CXCL13 in adaptive immune responses, we have reviewed the literature of CXCL13 in HIV/AIDS, focusing on its association with disease progression, the generation of broadly neutralizing antibodies (bnAbs) and HIV-associated B cell lymphoma.

## CXCL13 as a Biomarker in HIV-1 Infection

The plasma CXCL13 concentration has been reported to be elevated in HIV-1-infected individuals regardless of antiretroviral therapy (ART) treatment and disease stage (21–26). Plasma levels positively correlated with both HIV-1 disease progression and plasma levels of inflammatory markers, including CXCL10, soluble (s)CD23, sCD27 and sCD30 (23, 24). In a longitudinal study conducted in Canada, HIV-1-infected individuals sampled during both acute and chronic phases of infection showed higher plasma levels of CXCL13 compared to elite controllers and healthy controls (26). The increased CXCL13 levels were significantly reduced early after ART treatment but had not normalized after 24 months of treatment. This study also reported that the CD4 T cell count and CD4/CD8 ratio showed a significant inverse relationship with plasma levels of CXCL13 in HIV-1-infected individuals (26). The association of CXCL13 plasma levels to HIV disease progression even extends to mortality, as shown in a study conducted to assess risk factors for mortality in ART-treated HIV-1-infected adults. Additional plasma cytokine risk factors in this study included IL-6, sIL-2Rα and sCD14 (27).

Studies addressing quantitative serum or plasma CXCL13 measurements reported (**Table 1**), overall, show an increase of plasmatic CXCL13 levels in children and adults following HIV-1 infection; additional studies are, however, needed to assess CXCL13 levels longitudinally in seroconverted HIV-1 individuals with plasma specimens available prior to infection.

**Table 1:** Plasma or serum levels of CXCL13 in different clinical conditions.

	Children*	Adults#
Healthy controls	110.6 (75.1 – 169.6) pg/mL (28)	23 ± 13 pg/mL (23) 33.4 ± 14.9 pg/mL (26)
Early HIV-1 infection	NA	137.3 ± 67.4 pg/mL (26)
HIV-1 treated	226.9 (162.9 – 380.6) pg/mL (28)	112 ± 73 pg/mL (23)

NA, data not available; *, Median (IQR); #, Mean ± Standard deviation.

In a hepatitis B vaccination study in ART-treated HIV-1-infected adults, significantly elevated plasma levels of CXCL13 were measured in vaccine non-responders compared to responders; among responders, CXCL13 levels were negatively associated with anti-HBs levels, suggesting that pre-existing GC hyper-activation could impair vaccine responses during HIV-1 infection (29). In contrast, in an hepatitis B vaccination study of HIV-1-infected children, in which plasma CXCL13 levels were measured prior to vaccination and at 1 and 6 months after completion of the vaccination series, there was no significant change over time following vaccination in either HIV-1-infected or control children (21). Moreover, no correlation was found between plasma CXCL13 and hepatitis surface antibody (anti-HBs) levels at 1 month post-vaccination in either controls or HIV-1 infected individuals. However, as in the adult study, CXCL13 levels were higher at baseline in the HIV-1-infected children compared to control uninfected children.

With regard to specificity, immunization of mice with 4-hydroxy-3-nitrophenyl acetyl (NP) haptenated ovalbumin with aluminum hydroxide adjuvant also increased the plasma concentration of CXCL13, which reached a maximum level at 14 days after immunization. Moreover, plasma CXCL13 levels were associated with GC Tfh cells in line with the possibility that CXCL13 may have a general role as a biomarker for GC activities (14). With regard to mechanisms, a macaque study showing that plasma CXCL13 levels increased to a plateau 14 days after simian immunodeficiency virus (SIV) infection, suggested that the key steps occur upon or early after lentiviral infection (30). Consistent with this, a direct relationship between CXCL13 concentration and HIV-1 RNA copies in plasma was reported in HIV-1-infected children and adults (26, 28). There may be multiple sources of CXCL13 in patients; however, in cell-based studies, Cohen and colleagues showed that when blood cells were cultured in the presence of HIV-1, CXCL13 expression increased in monocytes but not in DCs or lymphocytes (24). The magnitude of increase was related to the concentration of HIV-1 ssRNA in culture media, suggesting the possibility that triggering the TLR7/8 pathway may be involved (24). Interferon (IFN)-α secretion by DCs was required for the monocyte CXCL13 response (24).

In addition to being triggered by HIV infection and serving as a potential biomarker, elevated circulating CXCL13 levels could significantly distort lymphocyte trafficking by increasing downregulation of CXCR5 (31). The above cited studies, taken together, suggest that dysregulation of CXCL13 expression during HIV-1 infection may be affected by multiple mechanisms, including levels of virus replication and HIV-1-induced inflammation.

## The Role of CXCL13 in the Development of Broadly Neutralizing Antibodies to HIV-1

Most vaccines or infections induce antibodies which can block or neutralize subsequent infection by the corresponding microorganism; however, antibodies produced during acute HIV-1 infection have limited impact in the control of viremia. Several years after the initial infection, few HIV-1-infected individuals produce antibodies able to neutralize *in vitro* a wide range of HIV-1 strains, so-called broadly neutralizing antibodies (bnAbs) (32). Isolation and characterization of bnAbs from HIV-1-infected subjects was made possible by cutting-edge molecular biology, and clinical trials have been conducted testing their therapeutic and prophylactic efficacy in both HIV-1-infected and healthy individuals, respectively (33, 34). Promising results were achieved with passive transfer of bnAbs to HIV-1-infected individuals, which have been shown to be safe and to reduce HIV-1 RNA in the circulation; eradication of the virus from latent reservoirs was, however, not possible with bnAbs alone (35). On the other hand, the combination of latency-reversing agents and bnAbs was able to induce provirus replication and to reduce the size of the virus reservoirs (35). As reviewed by Moore et al., generation of bnAbs is linked with higher HIV-1 viral load (36), suggesting that bnAbs may be a result of increased GC activities. In this regard, CXCL13 plasma levels have been shown to correlate with both neutralizing antibodies and circulating Tfh cell frequency (37). Importantly, in both early and chronic HIV-1 stages, plasma CXCL13 levels were significantly higher in broad neutralizers compared to narrow neutralizers (37), consistent with a role for Tfh and GC activity to promote somatic hypermutation and affinity maturation required for broad neutralizing activity. Among ART-naïve HIV-1-infected adults, top neutralizers with neutralization scores greater than 1 had higher plasma CXCL13 levels compared to low neutralizers with neutralization scores less than 0.5; the score was calculated based on the breadth and the potency of neutralizing antibodies in plasma (14).

Mabuka and colleagues showed that plasma levels of B- cell activation factor (BAFF) increased after 7 days from the onset of HIV-1 viremia and declined at 14 days, whereas, plasma levels of CXCL13 were elevated at day 7 from the onset of HIV-1 viremia and increased throughout the infection period (38). Additionally, plasma levels of CXCL13 in this study were significantly higher in the patients with detectable cross-neutralization activity compared to patients without detectable cross-neutralization activity at different time points during acute HIV-1 infection, suggesting a role for CXCL13 in predicting the emergence of cross-neutralization antibodies. This may reflect the role of Tfh-mediated affinity maturation in GCs in promoting production of cross-neutralizing antibodies. Plasma CXCL13 levels were associated with bnAbs at 6 months from HIV-1 infection but the correlation was lost after an additional 6 months (39). Fc polyfunctionality of bnAbs against gp120 peptides, measured by z-score of the Fc effector function, also correlated with plasma concentrations of CXCL13 (39). Dugast and colleagues studied inflammatory markers in HIV-1 controllers and the association of these molecules with bnAbs; among 18 markers, the serum levels of sCD40, IP-10, CXCL13, CCL5 and TNF-α were all able to predict the emergence of neutralizing antibody breadth (40). The study suggested that a group of markers could be useful to predict bnAbs activities instead of CXCL13 alone (40).

## CXCL13 in AIDS-Associated B Cell Lymphoma

CXCL13 has also been evaluated as a potential biomarker for early diagnosis of AIDS-associated non-Hodgkin B-cell lymphoma (41). Plasma CXCL13 levels were reported to be higher in this setting compared to HIV-1 positive controls without lymphoma during a 3-year follow-up period (41). In addition, plasma levels of CXCL13, IL-6 and IL-10 were potential markers for treatment outcomes and survival of patients with AIDS-associated non-Hodgkin lymphoma (NHL) (42).

## Future Directions

In addition to potential significance as a biomarker of GC function, increased plasma CXCL13 levels could have pathogenic significance during HIV-1 infection in view of their association with disease progression. Potential roles include driving the hyperinflammatory state and even contributing to the hypergammaglobulinemia seen in HIV infection. Overproduction of CXCL13 could also hinder effective vaccine responses and lead to poor generation of memory B cells by downregulating expression of its receptor, CXCR5. HIV-1 infected Tfh cells, both in tissue and in the periphery, are important reservoirs for the virus (43). It is possible that a high level of CXCL13 may contribute to survival of Tfh cells in the GC, thus contributing to persistence of HIV-1 reservoirs; this hypothesis is supported by the direct relationship observed between plasma CXCL13 concentration and HIV-1 RNA copies in HIV-1-infected patients (26, 28). In this regard, if validated as a therapeutic target, efforts to test anti-CXCL13 antibodies might be developed in an effort to reduce immune (especially B cell) activation.

## Conclusions

The studies reviewed here point to CXCL13 as a potential general biomarker for GC activity of particular interest in HIV/AIDS. In the context of vaccination, GC activities may reach a plateau at 2 weeks, so early measurements of plasma CXCL13 may help to further understand its role as a biomarker. The use of plasma CXCL13 as an adjunct to viral load for predicting the emergence of cross-neutralizing antibodies and bnAb during acute HIV-1 infection (and vaccines) should be investigated in a large cohort of HIV-1-infected patients.

## Author Contribution

YB: conception and design, review of the literature, preparing the figure, composition of the manuscript and final approval. FC, YS, and JAB: critical review of the literature, composition of the manuscript, and final approval. All authors contributed to the article and approved the submitted version.

## Funding

The work of FC is supported by a grant from the Swedish Medical Research Council (grant number 2019-01169) and intramural funding under project ZIA-C-004020 from the Center for Cancer Research, National Cancer Institute, National Institutes of Health, USA.

## Conflict of Interest

The authors declare that the research was conducted in the absence of any commercial or financial relationships that could be construed as a potential conflict of interest.
